# Interaction between V-ATPase B2 and (Pro) renin Receptors in Promoting the progression of Renal Tubulointerstitial Fibrosis

**DOI:** 10.1038/srep25035

**Published:** 2016-04-28

**Authors:** Yun Liu, Sujun Zuo, Xiaoyan Li, Jinjin Fan, Xueqin Cao, Xueqing Yu, Qiongqiong Yang

**Affiliations:** 1 Department of Nephrology, The First Affiliated Hospital of Sun Yat-Sen University, Guangzhou 510080, China; 2 Guangdong Provincial Key Laboratory of Nephrology, Guangzhou 510080, China

## Abstract

To investigate the levels of (Pro) renin receptor [(P) RR], α-smooth muscle actin (α-SMA), fibronectin (FN), and vacuolar H^+^-ATPase (V-ATPase) subunits (B2, E, and c) in rat unilateral ureteral obstruction (UUO) models and rat proximal tubular epithelial cells (NRK-52E) treated with prorenin to elucidate the role of V-ATPase in these processes by activating the (P) RR. UUO significantly upregulated (P) RR, V-ATPase subunits, α-SMA and FN expression in tubulointerstitium or tubular epithelial cells. A marked colocalization of (P) RR and the B2 subunit was also observed. Prorenin treatment upregulated α-SMA, FN, (P) RR, and V-ATPase subunits and activity in NRK52E cell in a dose- and time-dependent manner. The V-ATPase inhibitor bafilomycin A1 partially blocked prorenin-induced (P) RR, FN, and α-SMA expression. Co-immunoprecipitate and immunofluorescence results demonstrated that the V-ATPase B2 subunit bound to the (P) RR, which was upregulated after prorenin stimulation. Either siRNA-mediated (P) RR or B2 subunit knockdown partially reduced V-ATPase activity and attenuated prorenin-induced FN and α-SMA expression. From the data we can assume that activation of (P) RR and V-ATPase may play an important role in tubulointerstitial fibrosis with possible involvement of interaction of V-ATPase B2 subunit and (P)RR.

The renin-angiotensin system (RAS) is critical in the pathogenesis of the progression of chronic kidney disease (CKD)^1^. The prorenin receptor [(P) RR] is a novel component of RAS^2^. (P) RR activation plays an important role in the development of renal fibrosis by binding prorenin and renin, which are linked to angiotensin (Ang) II-dependent and Ang II-independent effects via the induction of intracellular signaling such as the ERK1/2 and p38 mitogen-activated protein (MAP) kinase pathways^3^,^4^,^5^. Several studies demonstrated that a peptide derived from the prorenin pro-segment (i.e., handle region peptide [HRP]) prevented the development of diabetic nephropathy and reduced cardiac fibrosis^6^,^7^,^8^, but other groups did not reproduce the positive effects of these peptides^9^,^10^. These discrepancies suggest the presence of unknown regulators of the biological function of (P) RR.

The (P) RR is composed of 350 amino acids in four different domains: a short cytosolic domain, a single transmembrane domain, an extracellular domain, and an N-terminal signal peptide^11^. The C-terminal (P) RR fragment is an accessory protein of the vacuolar-type H^+^-ATPase (V-ATPase)^12^. V-ATPase is a multi-subunit complex that contains two domains, namely, an ATP hydrolysis domain (V1) and a proton-pumping pore domain (V0), the latter of which is required for the translocation of protons across the membrane. The V1 domain in the mammalian counterpart is composed of eight different subunits (A-H), and the V0 domain consists of six different subunits, including a, c, c”, d, e, and the accessory subunit Ac45^13^. V-ATPase is responsible for the acidification of intracellular compartments and cellular pH homeostasis in all cell types. Vacuole acidification along these pathways is essential for many cellular functions including the processing of hormones such as insulin, receptor endocytosis and recycling, and membrane fusion events^13^. The treatment of several cell types with the V-ATPase inhibitor bafilomycin A1 produced morphological and pH changes that were similar to those in cells with a (P) RR deletion^14^,^15^. Bafilomycin prevented renin- and prorenin-induced ERK 1/2 phosphorylation in cultured collecting duct/distal tubule lineage Madin-Darby canine kidney cells^16^. Our previous study demonstrated that V-ATPase promoted TGF-β-induced epithelial-mesenchymal transition (EMT) and chronic tubulointerstitial fibrosis (TIF) by increasing V-ATPase activity via subunit redistribution or overexpression^17^. The roles of (P) RR, V-ATPase, and V-ATPase activity in the development and progression of renal fibrosis should be considered.

We hypothesized that V-ATPase is involved in the pathogenesis of TIF induced by prorenin via regulation of (P) RR and V-ATPase activity or subunit expression. We evaluated V-ATPase subunit and (P) RR expression and colocalization in an established animal model of kidney fibrosis, unilateral ureteral obstruction (UUO), and examined the roles and interactions of these proteins in prorenin-induced FN and SMA expression in NEK52E cells.

## Results

### Expression of (P) RR and V-ATPase Subunits after UUO

**Figure 1:**
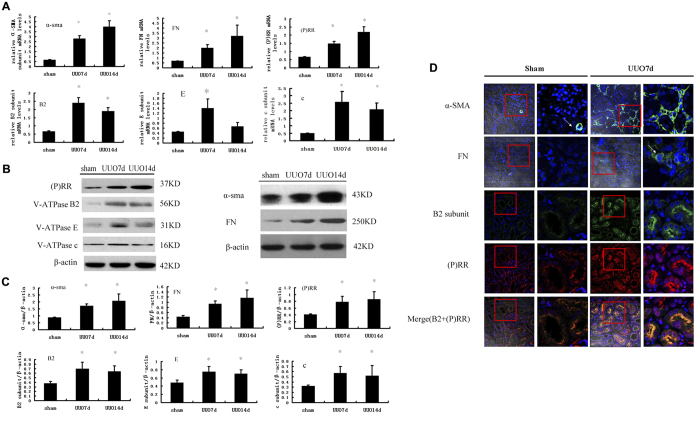
Expression of (P) RR, Vacuolar ATPase (V-ATPase) B2, E, c subunits, and α- SMA, fibronectin (FN) in kidney of SD rats at day 7 or 14 after UUO and SD rats with sham-operation. (**A**) Real-time PCR analysis showed mRNA expression of α-SMA, FN, (P) RR and Vacuolar ATPase (V-ATPase) B2, E, c subunits. Values are means ± SD; n = 4. *P < 0.05 vs. the sham group. (**B**) Western blot analysis of protein expression of (P) RR, V-ATPase subunits (B2, E, and c) and α- SMA, FN, showed higher expression of (P) RR, V-ATPase B2, E, c subunits, and α-SMA, FN in UUO rat. (**C**) Graphic representation of the protein levels of α-SMA, FN, (P) RR, V-ATPase B2, E, and c subunits at SD rats at day 7 or 14 after UUO and SD rats with sham-operation. Values are means ± SD; n = 4. *P < 0.05 vs. sham group. (**D**) immunofluorescence showed upregulation of (P) RR, V-ATPase B2, α-SMA and FN at SD rats kidney at day 7 after UUO respectively (×200), and the higher-magnification confocal microscopy images of the contents in red box (showed at right panel of Sham or UUO, ×400) showed that the B2 subunits and (P) RR significantly increased in apical region of tubular cells after UUO, and marked colocalization after UUO. Nucleus was stained with 4, 6-diamidino-2-phenylindole (blue). Confocal ×40 ocular.

Real-time PCR and western blotting analyses revealed that the expression of V-ATPase subunits (B2, E, and c) increased significantly in obstructed kidneys after UUO compared to sham-operated kidneys with higher expression of FN, α-SMA, and (P) RR (Fig. 1A–C). The peak expression of V-ATPase subunits occurred 7 days after UUO, which was earlier than the peak expression of FN, α-SMA, and (P) RR. Immunofluorescence demonstrated increased α-SMA and FN accumulation in the interstitial or tubular epithelial cells, and the V-ATPase B2 subunit and (P) RR dramatically accumulated in the apical regions of tubular epithelial cells 7 days after UUO (Fig. 1D). Dual-immunofluorescence staining using confocal microscopy revealed that (P) RR colocalized with V-ATPase B2, and it was markedly increased in the apical regions of tubular epithelial cells on day 7 after UUO (Fig. 1D).

### Prorenin-stimulated V-ATPase Subunit, (P) RR, and FN/α-SMA Expression in NRK52E Cells

**Figure 2:**
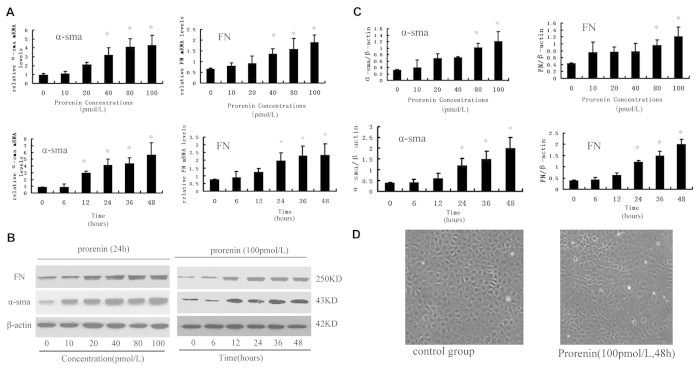
Prorenin stimulated FN and α-SMA expression of NRK52E cells with different concentrations (a: 0, 10, 20, 40, 80, or 100 pmol/L for 24 hours) or the different treatment times (b: 100 pmol/L for 0, 6, 12, 24, 36, or 48 hours). (**A**) Real-time PCR analysis showed mRNA expression of α-SMA and FN. Values are means ± SD; n = 4. *P < 0.05 vs. control group (0 hour or 0 pmol/L). (**B**) Western blot analysis of protein expression of α-SMA and FN. β-actin is an internal control. (**C**) Graphic representation of the protein levels of α-SMA and FN at different time or concentration points, as indicated after normalization with β-actin. Values are means ± SD; n = 4. *P < 0.05 vs. control group (0 hour or 0 pmol/L). (**D**) Phase-contrast images (magnification ×100) were captured to analyze cell morphology of NRK52E cells in control or prorenin stimulated group (100 pmol/L for 48 h).

We first treated NRK52E cells with prorenin to evaluate the expression of the fibrotic markers FN and α-SMA. NRK52E cell viability after prorenin treatment was examined using the MTT assay. Viability decreased after stimulation with 200 pmo/L prorenin for 24 h and 100 pmol/L prorenin for 72 h. Therefore, prorenin at 0, 10, 20, 40, 80, or 100 pmol/L for 24 h or 100 pmol/L for 0, 6, 12, 24, 36, or 48 h was used in the following studies. Fig. 2A–C shows that α-SMA and FN expression was markedly upregulated in a dose- and time-dependent manner after prorenin treatment. Cells treated with prorenin (100 pmol/L for 48 h) lost their cobblestone morphology and developed an elongated, fibroblast-like morphology (Fig. 2D).

**Figure 3:**
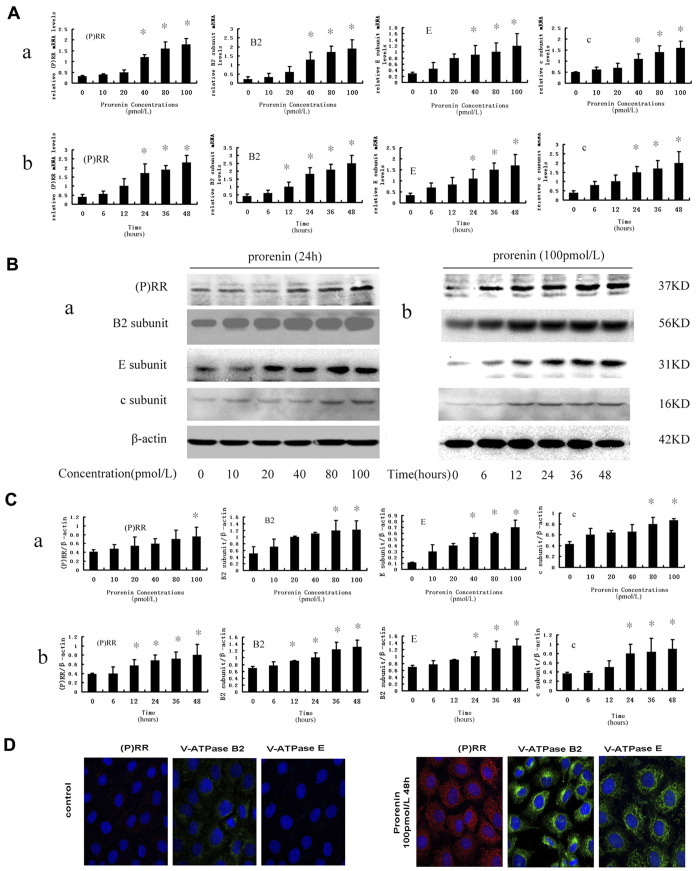
Expression of (P) RR, V-ATPase B2, E, and c subunit in NRK52E cells increased in a dose- and time-dependent manner after prorenin treatment. (**A**) Real-time PCR analysis showed mRNA expression of (P) RR, V-ATPase B2, E, and c subunit increased at a dose- (A-a) and time-dependent manner (A-b) after prorenin stimulation. Values are means ± SD; n = 4. *P < 0.05 vs. control group (0 hour or 0 pmol/L). (**B**) Western blot analysis showed the protein levels of (P) RR, V-ATPaseB2, E, and c subunits were also increased at a dose- (B-a) and time-dependent (B-b) manner. (**C**) Graphic representation of (P) RR, V-ATPase B2, E, and c protein levels in different groups (C-a showed as different dose treated and C-b as different times treated) as indicated after normalization with β-actin content. Values are means ± SD; n = 4. *P < 0.05 vs. control group (0 hour or 0 pmol/L). (**D**) Immunofluorescence staining demonstrated that the expression of (P) RR (red), V-ATPaseB2, E subunits (green) in NRK52E cells were significantly increased after stimulation with 100 pmol/L prorenin for 48 h. Nucleus was stained with DAPI (4, 6-diamidino-2-phenylindole) (blue). Confocal ×40 ocular.

We examined changes in (P) RR and V-ATPase expression in NRK52E cells stimulated with prorenin. Real-time PCR and Western blot analyses demonstrated that (P) RR and V-ATPase B2, E, and c subunits were normally expressed in NRK52E cells, and expression dramatically increased in a dose- and time-dependent manner after prorenin stimulation (Fig. 3A–C) that was identical to the trend in FN and α-SMA expression (Fig. 2A–C). Immunofluorescence staining confirmed these results and revealed (P) RR expression and the accumulation of V-ATPase B2 and E subunits with a diffuse cytoplasmic pattern in NRK52E cells following 48 h of 100 pmol/L prorenin stimulation (Fig. 3D).

**Figure 4:**
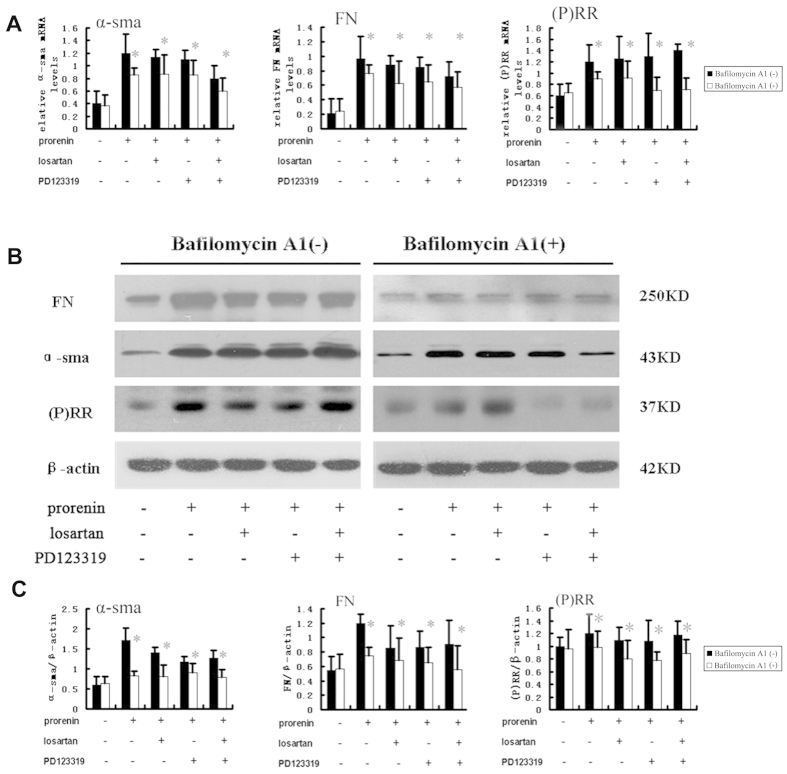
Effect of V-ATPase inhibitor bafilomycin A1 on FN, α-SMA and (P) RR expression in NRK52E cells after stimulated by prorenin (100 pmol/L) for 48 h. The cells were preincubated with or without the angiotensin II type1 receptor blocker losartan (10 μmol/L), the angiotensin II type2 receptor blocker PD123319 (10 μmol/L) or bafilomycin A1 (1 nmol/L) for 1 hour. Bafilomycin A1 partly inhibited expression of FN, α-SMA and (P) RR induced by prorenin. (**A**) Real-time PCR analysis showed mRNA expression of α-SMA, FN and (P) RR levels. (**B**) Western blot analysis of protein expression of α-SMA, FN and (P) RR in NRK52E cells by groups. (**C**) Graphic representation of the protein levels of α-SMA, FN and (P) RR. Values are showed with means ± SD; n = 4. *P < 0.05 Bafilomycin A1 (+) groups (showed as white bar) vs. Bafilomycin A1 (-) groups (showed as black bar).

We measured the mRNA and protein expression of (P) RR and α-SMA/FN using real-time PCR and western blotting in NRK52E cells stimulated by prorenin (100 pmol/L, 48 h) after pretreatment with bafilomycin A1 (1 nmol/L) to further evaluate the effect of the V-ATPase inhibitor bafilomycin A1 on prorenin-stimulated (P) RR and fibrotic marker (α-SMA/FN) expression in NRK52E cells (Fig. 4). The results demonstrated that bafilomycin A1 (1 nmol/L) partially blocked the expression of (P) RR and α-SMA/FN, which were upregulated by prorenin.

### Prorenin Stimulates V-ATPase Activity in NRK52E Cells

The B2, E, and c subunits of V-ATPase were upregulated after prorenin stimulation. We measured the hydrolysis activity of ATPase and the proton translocating activity of V-ATPase in NRK52E cells.

**Figure 5:**
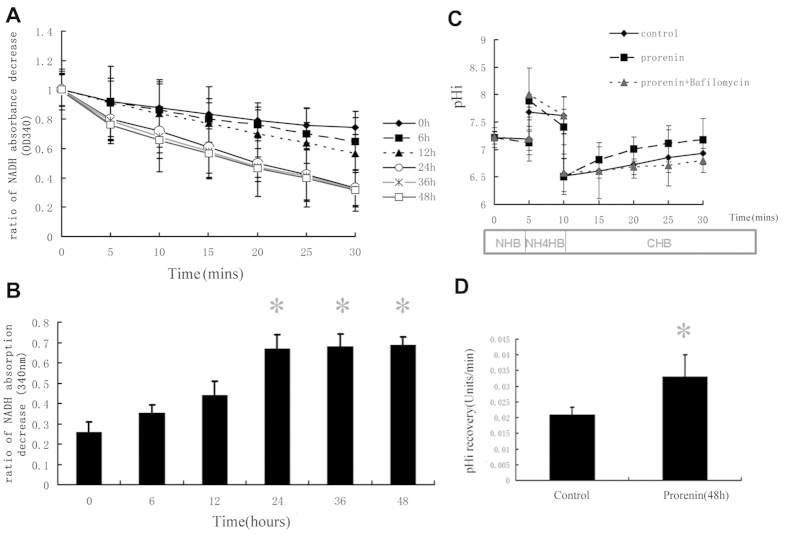
Effect of prorenin on hydrolysis activity of the ATPase and the proton-translocating activity of V-ATPase in NRK52E cells. The ATPase activity of the whole cell homogenates (60 μg) of NRK52E cells in different stimulated groups were measured by ATP/NADH-coupled assay. (**A**) Line graph showed as the results of ATP/NADH-coupled assay in different stimulated groups. (**B**) Bar graph showed the ratio of NADH absorption decrease at OD340 (ATP hydrolysis), and blanked against the cell lysis buffer (negative control). Control group (prorenin, 0 h) had measurable ATPase activity (0.259 ± 0.05 OD units/min, n = 6). After stimulation with prorenin, ATPase activity was increased significantly on 24, 36 and 48 h (0.670 ± 0.07, 0.681 ± 0.06, 0.688 ± 0.04 OD units/min, respectively P < 0.05, n = 6). Values are with means ± SD; *P < 0.05 vs. Control group. The Na^+^-independent intracellular pH (pHi) recovery after acute cellular acidification methods was measured as the proton-translocating activity of V-ATPase. (**C**) Cell pHi was determined with a fluorescence probe SNARF-4F. NRK 52E cells were bathed in a 110 mM Na^+^ solution (NHB), in which the basal pHi was 7.21 ± 0.12 (n = 6). After 5 min of exposure to NH_4_Cl, during which pHi increased transiently to 7.68 ± 0.32 (n = 6), NH_4_Cl removal caused a rapid acidification of pHi (6.51 ± 0.33, n = 6) as a result of NH_3_ efflux. Subsequently, the effect of a Na^+^-free solution (CHB) on the pH recovery of NRK52E cells, which was mainly mediated by V-ATPase, was depicted in the control groups (♦), prorenin-stimulated groups (■), and prorenin-stimulated in the presence of bafilomycin A1 groups (▲). (**B**) The rate of pHi recovery of control and prorenin stimulated group. Values are means ± SD. *P < 0.05 vs. control.

The ATPase activity of NRK52E cells stimulated with 100 pmol/L prorenin was determined at different time points using the ATP/NADH-coupled assay. ATPase activity increased with time after prorenin stimulation, and the results in Fig. 5A,B show a significant increase after 24 h. NADH absorption at OD340 (ATP hydrolysis) increased (0.670 ± 0.07, 0.681 ± 0.06, and 0.688 ± 0.04 at 24, 36, 48 h, respectively, vs. 0.259 ± 0.05 OD units/min for the control, P < 0.05, n = 6).

The proton translocating activity of V-ATPase in NRK52E cells was measured as Na^+^-independent intracellular pH recovery following an acute acid load using the NH_4_Cl pulse technique (Fig. 5C,D). The pHi recovery rate was 0.021 ± 0.002 pH U/min in the control group (n = 6). The pHi recovery rate after stimulation with 100 pmol/L prorenin for 48 h was significantly increased (0.033 ± 0.007 pH U/min, n = 6) compared to the control (P < 0.05). The V-ATPase inhibitor bafilomycin (1 μM) nearly abolished this increase, with a decrease in pHi recovery rate to 0.011 ± 0.003 pH U/min (n = 6).

### Interaction of the V-ATPase B2 subunit and (P) RR

**Figure 6:**
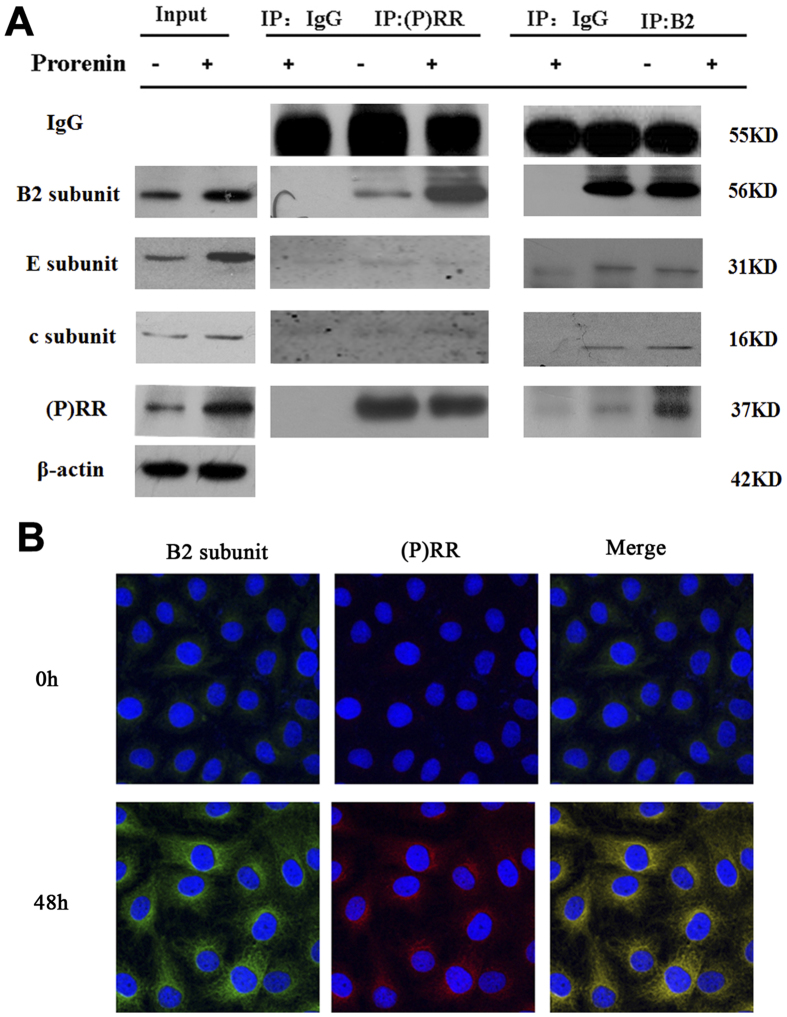
Interaction between (P) RR and V-ATPase B2 by co-immunoprecipitates and co-immunostaining. (**A**) 800 μg of whole cell homogenates from NRK-52E cells stimulated by prorenin (100 pmol/L, 0 and 48 h) were prepared with 4 μg antibodies or 2 μg IgG as a negative control. Western blots demonstrating the presence of the V-ATPase B2 subunit (56KD) in anti-(P) RR (IP: (P) RR, Rabbit polyclonal antibody) immunoprecipitates, and this effect was upregulated after prorenin stimulation (100 pmo/L, 48 h); meanwhile, the presence of the (P) RR (37KD) and other subunits of V-ATPase (E subunits, 31KD; c subunits, 16 kD) were measured by Western blots after immunoprecipitated by anti-V-ATPase B2 subunit (IP: B2, mouse monoclonal antibody), and this effect was also upregulated after prorenin stimulation (100 pmo/L, 48 h). (**B**) Co-immunofluorescence results showed marked colocalization of (P) RR (red) and vacuolar ATPase B2 subunit (green) in NRK52E cells after prorenin stimulation. Nucleus was stained with 4,6-diamidino-2-phenylindole (blue). Confocal ×40 ocular.

The B2 subunit markedly accumulated with (P) RR in the UUO models. Therefore, we examined the interaction of the V-ATPase B2 subunit and (P) RR in NRK52E cells. Co-immunoprecipitation results demonstrated that (P) RR significantly bound to the V-ATPase B2 subunit, but not the E and c subunits, and this effect was significantly increased after prorenin stimulation (100 pmol/L, 48 h) (Fig. 6A). Co-immunofluorescence staining and confocal microscopy also confirmed that (P) RR colocalized with V-ATPaseB2, which increased markedly following prorenin stimulation (Fig. 6B).

### Down regulation of (P) RR or the V-ATPase B2 subunit using siRNA-Mediated Silencing reduced V-ATPase Activity and fibrotic marker expressioninNRK52ECells stimulated by prorenin

The above results demonstrated that (P) RR bound to the V-ATPase B2 subunit and that the expression increased significantly after prorenin stimulation. NRK52E cells were transfected with B2 isoform-specific siRNA or (P) RR-specific siRNA to reduce the level of B2 or (P) RR, followed by a 48-h incubation with prorenin to further demonstrate the role of the interaction of (P) RR and the V-ATPase B2 subunit in the modulation of prorenin-induced fibrotic factor expression.

**Figure 7:**
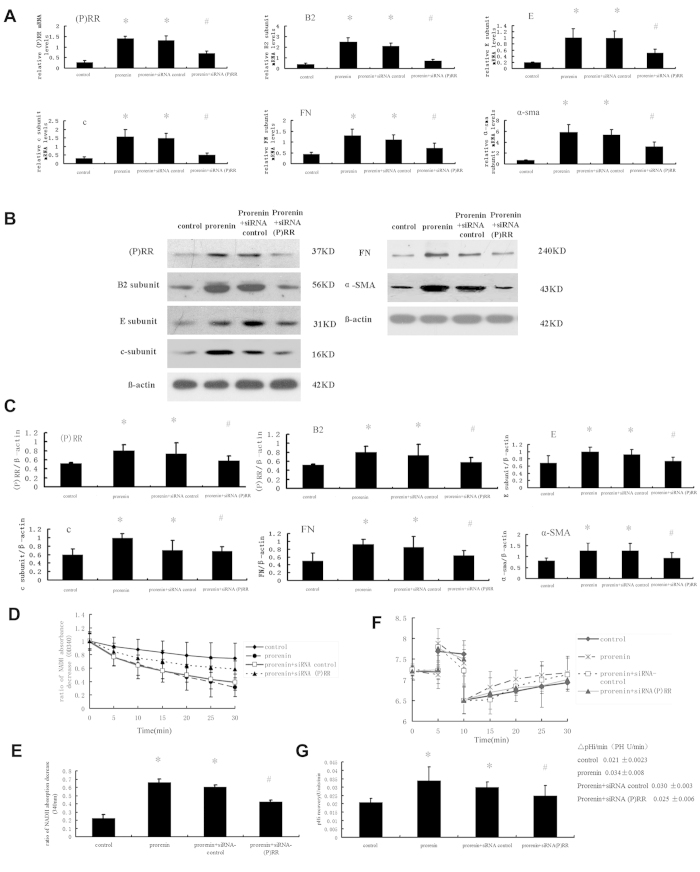
Effects of small interfering (si) RNA-mediated silencing of (P) RR on expression of fibrosis marker (FN and α-SMA), V-ATPase subunits (B2, E, and c) and activity in NRK52E cells after stimulation by prorenin (100 pmol/L) for 48 h. Cells were divided into the four groups as follows: control group, prorenin-treated group, prorenin-treated groups transfected with non-silencing siRNA (siRNA-control) or specific (P) RR siRNAs (siRNA- (P) RR). (**A**) mRNA levels of (P) RR, V-ATPase subunits (B2, E, and c), and fibrosis marker (FN and α-SMA) in NRK52E cells by groups. Values are with means ± SD; n = 6; *P < 0.05 vs. control group, #P < 0.05 vs. prorenin-treated group. (**B**) Western blots analysis of (P) RR, V-ATPase subunits (B2, E, and c), and fibrosis marker (FN and α-SMA) in NRK52E cells by groups. (**C**) Graphic representation of the protein levels. Values are with means ± SD; n = 6; *P < 0.05 vs. control group, #P < 0.05 vs. prorenin-treated group. (**D**) Line graph showed as the results of ATP/NADH-coupled assay in different stimulated groups. (**E**) Bar graph showed the ratio of NADH absorption decrease at OD340 (ATP hydrolysis). Compared to the prorenin-treated group, the ATPase activity in the siRNA-(P) RR group was lower (0.420 ± 0.019 vs. 0.664 ± 0.04 OD units/min), n = 6; #P < 0.05 vs. prorenin-treated group *P < 0.05 vs. control group. (**F**) The Na^+^-independent intracellular pH (pHi) recovery after acute cellular acidification methods was measured as the proton-translocating activity of V-ATPase by groups. The effect of a Na^+^-free solution (CHB) on the pH recovery of NRK52E cells, which was mainly mediated by V-ATPase, was depicted in the control groups (♦), prorenin-stimulated groups (*), and prorenin-treated group transfected with non-silencing siRNA (siRNA-control) (□) or specific (P) RR siRNAs (▲). (**G**) The rate of pHi recovery of different groups. Values are with means ± SD; n = 6. *P < 0.05 vs. control group, #P < 0.05 vs. prorenin-treated group.

SiRNA-based (P) RR knockdown efficiency was evaluated using immunoblotting and confirmed using densitometry analyses (Fig. 7A–C). A greater than 50% reduction in (P) RR levels was observed in cells treated with (P) RR siRNA followed by a 48-h treatment with prorenin. The specificity of (P) RR siRNAs for (P) RR knockdown was confirmed in NRK52E cells transfected with non-targeting siRNA (siRNA-control). Real-time PCR and immunoblotting confirmed (P) RR expression in (P) RR knockdown and siRNA control cells.

Down-regulation of (P) RR reduced prorenin-induced fibrosis marker (α-SMA and FN) and V-ATPase subunit (B2, E, and c) expression (Fig. 7A–C). Prorenin stimulation increased the ATPase activity of V-ATPase (0.664 ± 0.04 vs. 0.228 ± 0.04 OD units/min for the prorenin groups vs. control groups, respectively, P < 0.05, n = 6). SiRNA-mediated silencing of (P) RR partially inhibited the increased activity (0.420 ± 0.019 OD units/min) (Fig. 7D,E). (P) RR-specific siRNAs, but not non-targeting siRNA, markedly decreased the prorenin-stimulated increase in V-ATPase proton translocating activity, as demonstrated by the reduced Na^+^-independent pHi recovery following an acute acid load (0.025 ± 0.006 pH U/min for siRNA (P) RR-treated groups vs.0.034 ± 0.008 pH U/min for prorenin-stimulated groups, or 0.030 ± 0.003 pH U/min for prorenin and non-targeting siRNA-treated groups, n = 6, P < 0.05) (Fig. 7F,G).

**Figure 8:**
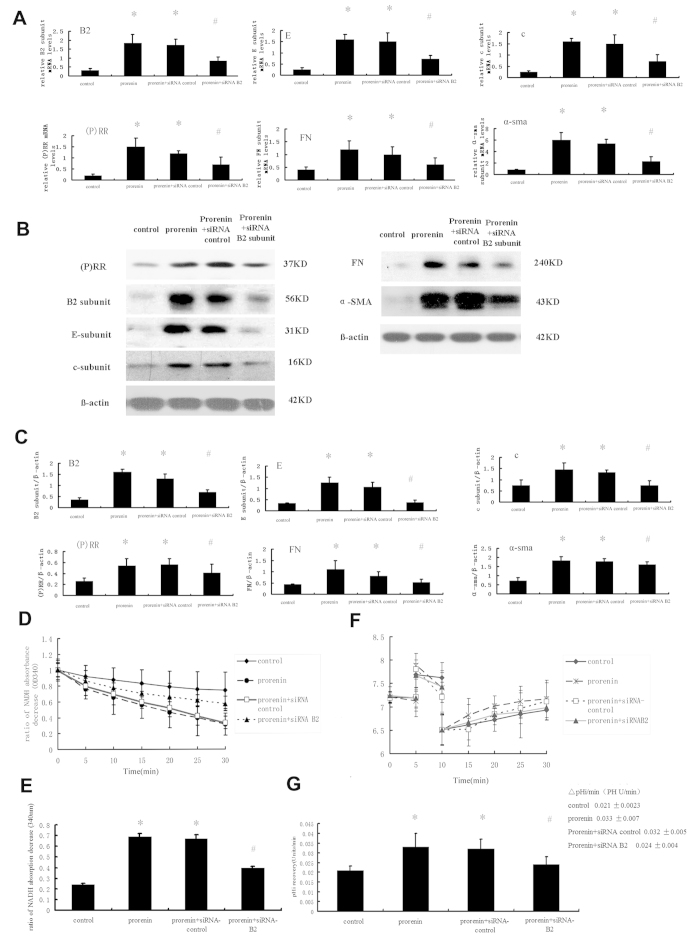
Effects of small interfering (si) RNA-mediated silencing of V-ATPase B2 subunit on expression of fibrosis marker (FN and α-SMA), (P) RR, and other V-ATPase subunits (E and c), and V-ATPase activity in NRK52E cells after stimulation by prorenin (100 pmol/L) for 48 hours. Cells were divided into the four groups as follows: control group, prorenin-treated group, prorenin-treated group transfected with non-silencing siRNA (siRNA-control) or specific V-ATPase B2 subunit siRNAs (siRNA-B2). (**A**) mRNA levels of (P) RR, V-ATPase subunits (B2, E, and c), and fibrosis marker (FN and α-SMA) in NRK52E cells by groups. Values are with means ± SD; n = 6; *P < 0.05 vs. control group, #P < 0.05 vs. prorenin-treated group. (**B**) Western blots analysis of (P) RR, V-ATPase subunits (B2, E, and c), and fibrosis marker (FN and α-SMA) in NRK52E cells by groups. (**C**) Graphic representation of the protein levels. Values are with means ± SD; n = 6; *P < 0.05 vs. control group, #P < 0.05 vs. prorenin-treated group. (**D**) Line graph showed as the results of ATP/NADH-coupled assay in different stimulated groups. (**E**) Bar graph showed the ratio of NADH absorption decrease at OD340 (ATP hydrolysis), and compared to the prorenin treated group, ATPase activity in the siRNA-B2 group was lower (0.400 ± 0.013 vs. 0.689 ± 0.03 OD units/min, P < 0.05). Values are presented as means ± SD; n = 6; *P < 0.05 vs. control group, #P < 0.05 vs. prorenin-treated group. (**F**) The Na^+^-independent intracellular pH (pHi) recovery after acute cellular acidification methods was measured as the proton-translocating activity of V-ATPase by groups. The effect of a Na^+^-free solution (CHB) on the pH recovery of NRK52E cells, which was mainly mediated by V-ATPase, was depicted in the control groups (♦), prorenin-stimulated groups (*), and prorenin-treated group transfected with non-silencing siRNA (siRNA-control) (□) or specific V-ATPase B2 subunit siRNAs (▲). (**G**) The rate of pHi recovery of different groups. Values are with means ± SD; n = 6. *P < 0.05 vs. control group, #P < 0.05 vs. prorenin-treated group.

Similarly, the effect of siRNA-based B2 knockdown on the modulation of fibrotic factors (FN or α-SMA) and prorenin-stimulated (P) RR expression was also evaluated. Figure 8A–C shows that the down-regulation of the B2 subunit reduced the expression of prorenin-stimulated fibrosis markers (α-SMA and FN), other V-ATPase subunits (E and c), and (P) RR. B2 siRNA partially inhibited the prorenin-stimulated increase in ATPase activity of V-ATPase (0.400 ± 0.013 vs. 0.689 ± 0.030 OD units/min for B2 siRNA-treated groups and the prorenin-treated group, respectively, P < 0.05, n = 6) (Fig. 8D,E). The introduction of specific V-ATPase B2 subunit siRNAs markedly decreased the prorenin-stimulated increase in the proton translocating activity of V-ATPase in NRK52E cells (i.e., the rate of Na^+^-independent pHi recovery following an acute acid load in cells: 0.024 ± 0.004 pH U/min for siRNA B2-treated groups vs. 0.033 ± 0.007 pH U/min for prorenin-treated groups, n = 6, P < 0.05) (Fig. 8F,G).

## Discussion

The present study provided evidence of a role of V-ATPase in the (P) RR regulation of prorenin-induced TIF-associated changes in tubular epithelial NRK52E cells and indicated that the interaction of (P)RR and B2 subunit of V-ATPase may play an important role in this process. First, we found that (P) RR and V-ATPase subunits were increased in renal tissue of a UUO rat model and that (P) RR markedly colocalized with the V-ATPase B2 subunit, with a significant accumulation in the apical region of tubular epithelial cells. The expression of (P) RR and V-ATPase subunits and V-ATPase activity increased with fibrotic factor (α-SMA/FN) upregulation in NRK52E cells treated with prorenin. These findings were further confirmed using the specific V-ATPase inhibitor bafilomycin A1, which attenuated (P) RR and fibrotic factor expression in NRK52E cells following prorenin treatment. Co-immunofluorescence and co-immunoprecipitation revealed the interaction between (P) RR and the V-ATPase B2 subunit. siRNA-mediated specific knockdown of (P) RR or the B2 subunit partially reduced the V-ATPase activity and attenuated FN and α-SMA expression induced by prorenin. These results further suggest that V-ATPase plays a role in the modulation of (P) RR-regulated TIF by partially affecting the proton transport or binding ability of the V-ATPase complex to (P) RR via the B2 subunit.

(P) RR activation plays an important role in the development of renal fibrosis^18^. (P) RR specifically binds renin and prorenin with the following two important consequences: enzymatic activation of prorenin and enhancement of tissue RAS. (P) RR activation also induces intracellular signaling pathways. Tissue RAS activation is an important process in renal fibrosis^19^. The binding of prorenin triggers intracellular signaling and the activation of ERK1/2 MAP kinases, which leads to the upregulation of TGF-1, PAI-1, collagens, fibronectin^3^,^4^,^20^, and cyclooxygenase-2^21^. Transgenic animals over-expressing (P) RR ubiquitously or selectively in smooth muscle cells develop high blood pressure^22^ or glomerulosclerosis^8^. A strong increased expression of (P) RR was reported in UUO nephrotubulus compared to contralateral unobstructed kidney (CUK) nephrotubulus^23^. Our study also revealed a higher expression of (P) RR in the kidneys of UUO rats, and (P) RR increased with FN and α-SMA increases in a dose- and time-dependent manner in prorenin-treated NRK52E cells after pretreatment with the Ang II type 1 receptor blocker losartan and the Ang II type 2 receptor blocker PD 123319. These results confirmed that (P) RR induced renal interstitial fibrosis via RAS-independent pathways.

In this study, we found prorenin upregulated (P) RR and V-ATPase with increasing with fibrotic factors (α-SMA/FN) in NRK52E cells. Using the specific V-ATPase inhibitor bafilomycin A1 attenuated (P) RR and fibrotic factor expression in NRK52E cells following prorenin treatment, supporting the role of V-ATPase in prorenin activation of (P)RR in the TIF process. This is an unexpected finding since prorenin seems to bind (P)RR, a receptor for (pro)renin, and lead to the activation of tissue renin-angiotensin system and intracellular signalings. However, it is known that (P)RR also plays an important role as V-ATPase associated protein, involving in Wnt signaling^24^ and V-ATPase assembly and function^14^. These may possibly promote more full-length and truncated (P)RR translocate to plasma membranes and increase the (P)RR in membranes. Additional studies are needed to confirm this hypothesis.

Meanwhile, the (P) RR research field has contributed to an increased understanding of the role of V-ATPase in local RAS and receptor activation by the surprising identification of ATP6AP2/(P) RR as an accessory subunit of V-ATPase. Our study also revealed that the expression of V-ATPase subunits (B2, c, and E) and ATPase and the proton translocating activity of V-ATPase were upregulated during the process of prorenin-induced fibrosis, and the expressions of (P) RR and V-ATPase subunits peaked earlier than those of fibrotic factors did. These results suggest that activated V-ATPase participated in the process of prorenin-induced fibrosis and that V-ATPase may act as an upstream regulator. V-ATPase is a holoenzyme that consists of a multi-subunit complex containing a V1 domain and V0 domain. Our study revealed that the E and B2 subunits of the V1 domain and the c subunit of the V0 domain increased along with the increase in (P) RR-regulated prorenin-induced FN and α-SMA expression and V-ATPase holoenzyme activity. The V-ATPase inhibitor bafilomycin A1 partially reduced (P) RR, α-SMA, and FN expression in NRK52E cells stimulated by prorenin. These results demonstrated the role of V-ATPase as a holoenzyme during this process. Our previous studies also suggested that V-ATPase participated in TGF-β-induced EMT as an intact enzyme by influencing intracellular acidification^17^. Intracellular acidification is an early event in the apoptosis process, and V-ATPase plays an important role in the regulation of apoptosis (e.g., cancer cells, neutrophils)^25^,^26^. Our results suggest a novel role for V-ATPases in the process of (P) RR-regulated renal fibrosis through intracellular pH regulation, but additional studies are still necessary to elucidate the exact mechanisms of V-ATPases in TIF.

Our study also demonstrated that the B2 subunit of V-ATPase markedly colocalized with (P) RR in the UUO model and prorenin-stimulated NRK-52E cells. Co-immunoprecipitation further confirmed that (P) RR bound to the V-ATPase B2 subunit, but not the E and c subunits. Binding increased significantly after prorenin stimulation for 48 h. Notwithstanding, the RNAi-mediated specific knockdown of B2 or (P) RR demonstrated that specific knockdown of B2 or (P) RR subunits partially attenuated prorenin-induced FN and α-SMA expression via the downregulation of V-ATPase activity. These data suggest that the interaction between the V-ATPase B2 subunit and (P) RR is involved in the process of TIF. The 56-kD B subunit of V-ATPase is part of the catalytic core, and it possesses regulatory functions in V-ATPase. The B subunit as two highly homologous isoforms, B1 (ATP6 V1B1) and B2 (ATP6 V1B2). The B2 subunit is ubiquitously expressed, and it may be responsible for the translocation of V-ATPase from the cytosol to the membrane. Carraro-Lacroix *et al.* demonstrated that immortalized rat proximal tubular cells exposed to angiontensin II exhibited an upregulation of V-ATPase activity that was partially caused by increased B2 cell surface expression; the B2 subunit contains an F-actin binding site, which interacts with actin filaments and allows trafficking to the cell surface^27^. The B2 subunit was upregulated in V-ATPase B1 subunit-deficient mice to compensate for the lack of B1, which was sufficient to maintain basal acid-base homeostasis, despite the downregulation of other V-ATPase subunits in the animal model^28^. The V-ATPase B2 subunit plays an important role in the regulation of V-ATPase in addition to forming the enzyme complex for proton transport. V-ATPase is indispensable for the normal function of endosomes, lysosomes, and autophagosomes, and (P) RR is a multi-functioning protein, serving as an accessoryprotein of V-ATPase and the Wnt receptor complex^24^. Therefore, the overexpression of (P) RR may yield adverse effects through (P) RR-distinctive signal transductions, Ang II-mediated effects, and other injurious molecules, which contribute to the progression of renal fibrosis. The B2 subunit may specifically mediate or trigger V-ATPase interactions with (P) RR. Future experiments are needed to investigate the pathophysiological functions of (P) RR in V-ATPase activity and signaling aspects in renal fibrosis. Synthetic HRP, a peptidergic antagonist of (P) RR, blocks the combination of prorenin and (P) RR, but experimental results are controversial^6^,^7^,^8^,^9^,^10^. HRP was designed based on the idea that the prosegment of prorenin contains a ‘handle region’ (10P-19P) that competitively binds to the receptor to prevent receptor-mediated prorenin activation and reduce tissue RAS activity^29^. Findings suggest that HRP attenuates the development of diabetic nephropathy in diabetic mice^6^ and cardiac fibrosis in SHRsp rats in an Ang II-dependent or -independent manner^7^. However, whether HRP effects are truly due to an interference of the (pro)renin-(P) RR interaction *in vivo* is not known^9^,^10^. Other studies revealed no effects of HRP on blood pressure, cardiac hypertrophy, or renal damage when HRP was infused in 2-kidney 1-clip Goldblatt rats^30^. HRP did not prevent (pro) renin signaling in monocytes^31^ or vascular smooth muscle cells^32^ HRP increased the activation of the phosphorylated proliferation-related signaling pathway ERK1/2^33^, which is associated with neuronal and glial injury in the retina^34^. These controversies suggest that (P) RR is not located on the cell membrane in some situations, which may prevent the easy access of HRP to the receptor. Our study demonstrated that activated V-ATPase promoted (P) RR expression and prorenin-induced fibrosis, which suggests that V-ATPase regulates (P) RR and V-ATPase activity and that these factors should be considered in the HRP-blocking process.

The role of B2 in prorenin-induced fibrosis is also interesting. Our study demonstrated that B2 interacted with (P) RR and that siRNA-mediated B2 subunit knockdown partially reduced prorenin-induced V-ATPase activity and attenuated FN and α-SMA expression. These results suggest that the interaction of B2 with (P) RR contributes to (P) RR mediation and V-ATPase activation. However, B2 is also associated with V-ATPase-independent functions. Our previous study demonstrated that the free-unassembled B2 subunit is a cell survival factor that inhibits apoptosis via activation of the Ras-MAPK signaling pathway without increasing V-ATPase activity^35^. Our group also recently found that TGF-β1 induced cell surface expression of the B2 subunit in normal rat kidney epithelial (NRK52E) cells and that siRNA-mediated B2 subunit knockdown partially reduced TGF-β1-induced V-ATPase activity and attenuated EMT^17^. These two studies demonstrate that the V-ATPase B2 subunit exerts important effects in renal fibrosis and that it interacts with (P) RR. Therapeutic targets in prorenin-induced fibrosis should focus on the B2 subunit in the future.

Several questions remain unaddressed by this current study and require further investigation. Whether the combination of siRNA-assisted down-regulation of the V-ATPase B2 subunit with HRP treatment would further decrease the expression level of (P) RR and fibrotic markers in NRK52E cells is not known. Studies using animal models would be required to ascertain whether the interaction between (P) RR and the V-ATPase B2 subunit that we observed in *in vitro* experiments would similarly regulate the activity of V-ATPase *in vivo*. It was reported that the (pro)renin concentrations required to activate intracellular signaling *in vitro* may be much higher than the (patho)physiological plasma levels^36^,^37^ Plasma levels of (pro)renin in UUO rat were not assessed in this study, which could be limited our interpretation to the findings on NRK-52E cells stimulated by prorenin. However, the use of UUO rats in this present study may complicate the interpretation of the results because the process of renal fibrogenesis in this animal model may be affected by inflammatory factors such as IL-1, TNF-α, and IFN-γ^38^. Therefore, it may be necessary to verify our findings in human (P) RR-transgenic rats. Furthermore, detailed structural information on the domain architectures of (P) RR and V-ATPase is needed to provide a mechanistic rationale for the interaction between (P) RR and the V-ATPase B2 subunit. In summary, this study suggests an involvement of V-ATPase in TIF-associated cell changes induced by the activation of (P) RR, which may be related to V-ATPase activity and the binding of the V-ATPase B2 subunit to (P) RR. These findings also propose that the V-ATPase B2 subunit may be a potential drug target that impedes TIF-associated changes. Further research is necessary to decipher the domain architectures of (P) RR and the V-ATPase B2 subunit and better understand the mechanisms of their interaction.

## Methods

### Ethics statement

All animal experiments were approved by the Animal Care and Use Committee of Sun Yat-sen University ethnically. The methods were carried out in accordance with the approved guidelines. All experimental protocols were approved by Guangdong Provincial Key Laboratory of Nephrology in the First Affiliated Hospital of Sun Yat-Sen University. All methods were performed in accordance with the approved guidelines.

### Animal Studies

Male Sprague-Dawley rats, weighing 200–250 g, were housed in the animal care facility at Sun Yat-sen University (Guangzhou, China). The UUO procedure was performed as described previously^17^,^39^. Sham-operated rats (*n* = 4) were used as controls. All experiments adhered to the guidelines of the Animal Care and Use Committee of Sun Yat-sen University. Animals were sacrificed 7 and 14 days (*n* = 4) after UUO, and both kidneys were harvested. One kidney was formalin-fixed and paraffin-embedded for immunohistochemical analyses. The other kidney was immediately stored in liquid nitrogen for protein or mRNA collection.

### Cell Culture

NRK-52E cells (ATCC, Manassas, VA) were cultured and prepared as previously described^17^. Cells were treated with human recombinant prorenin (Cayman Chemical, Ann Arbor, MI) at 0, 10, 20, 40, 80, and 100 pmol/L for 24 h or 100 pmol/L for 0, 6, 12, 24, and 48 h after preincubation with the Ang II type 1 receptor blocker losartan (10 μmol/L) (Sigma, San Francisco, CA,) or the Ang II type 2 receptor blocker PD123319 (10 μmol/L) (Sigma, San Francisco, CA) for 1 h.

The cells were treated with bafilomycin A1 (1 nmol/L, Sigma-Aldrich), losartan (10 μmol/L), and PD123319 (10 μmol/L) for 1 h before prorenin (100 pmol/L) incubation for 48 h to evaluate the effects of the V-ATPase inhibitor bafilomycin A1 on prorenin-induced FN and α-SMA expression in NRK52E cells. Cells were harvested and used in MTT (3-(4, 5-dimethyl (thiazol-2-yl)-2, 5-diphenyltetrazolium bromide) cell viability, immunoblotting, real-time polymerase chain reaction (PCR), and immunofluorescence assays.

### Western Blot Analysis

Immunoblotting was performed as previously described^17^, using a lysis buffer (Cell Signaling Technology, Boston, MA) with a protease inhibitor cocktail (Cell Signaling Technology, Boston, MA). Proteins (50 μg) were loaded in 7%, 10%, and 15% sodium dodecyl sulfate-polyacrylamide gel electrophoresis (SDS-PAGE) (determined by molecular weight). The following primary antibodies were used: mouse anti-α-SMA (1:500, Millipore, Boston, MA), mouse anti-β-actin (1:5,000, Sigma, St. Louis, MO), mouse anti-fibronectin (FN) (1:1000, Cell Signaling Technology, Boston, MA), mouse anti-V-ATPase B2 (1:500, SantaCruz Biotechnology, SantaCruz, CA), rabbit polyclonal anti-V-ATPase E(1:500, Abcam, Cambridge, UK), rabbit polyclonal anti-V-ATPase c (1:250, Millipore, Billerica MA), rabbit polyclonal anti-(P) RR (1:200, SantaCruz Biotechnology, SantaCruz, CA), and sheep polyclonal anti-renin/(pro) renin (1:2000, Cell Science, Canton, MA). The image analysis program FluorChem8900 (AlphaInnotech) was used for densitometric analyses of positive immunoreactive bands, and the results were normalized to β-actin and compared to the control. The results are displayed as representative assays of four sets of independent experiments.

### RNA Extraction, Purification, and Real-Time PCR Analyses

Total RNA was extracted from NRK52E cells or rat kidneys using TRIzol reagent (Invitrogen) according to the manufacturer’s protocol. RNA concentration measurements and cDNA acquisition were performed following the protocol of our previous study^17^, The cDNA was used in real-time PCR. Invitrogen synthesized the primers (Table 1). A total volume of 10 μL of the mixture, including cDNA (1 μL for one reaction cycling), primer (0.5 μL), DEPC-treated water (3.5 μL), and SYBR Green master (Roche Molecular Biochemicals, Indianapolis, IN, 5 μL), was amplified in anABI7900HT real-time PCR system (Applied Biosystems, Foster City, CA). The following reaction cycling conditions were used: 2 min at 50 °C, 10 min at 95 °C for enzyme activation, and 40 cycles of 15 s at 95 °C for denaturation and 1 min at 58 °C for annealing and extension. The fluorescence threshold value was calculated using the SDS 7000 software (Applied Biosystems). Relative gene expression data were analyzed using the 2^−ΔΔCT^ method with normalization to β-actin. Experiments were performed in triplicate.

### Immunostaining

#### Tissue sections

Paraffin-embedded kidney sections (6-μm thick) were obtained from UUO and sham-operated rats for immunofluorescence. The immunofluorescence staining was performed following procedures described in our previous study^17^. Tissue sections were stained with an anti-α-SMA (1:50, Millipore, Boston, MA) or anti-FN (1:50, BD, Franklin Lake, NJ) antibody overnight at 4 °C and double-stained with anti-V-ATPase B2 (1:50, Santa Cruz Biotechnology, Santa Cruz, CA) and anti-(P) RR (1:50, Abcam, Cambridge, UK) antibodies.

#### Cell culture studies

NRK52Ecells were cultured on15 × 15-mm coverslips in 35-mm plates and treated with or without prorenin (100 pmol/L, 48 h). Indirect immunofluorescence staining was performed as described in our previous study^17^. Cells were stained using anti-V-ATPase E subunit (1:25, Abcam, Cambridge, UK), anti-(P) RR (1:50, Abcam, Cambridge, UK), or anti-V-ATPase B2 (1:50, Santa Cruz, CA) antibodies or double-stained with anti-(P) RR and anti-V-ATPase B2 antibodies, followed by staining with anti-mouse Alexa Fluor 546, or anti-mouse Alexa Fluor 488, or anti-rabbit Alexa Fluor 488antibodies (1:1,000, Invitrogen). Cells were counterstained with 4, 6-diamidino-2-phenylindole (DAPI) (1:200, Invitrogen) for 7 min to identify nuclei. Coverslips were secured using Fluoro-gel with Anti-Fading Agent (Electron Microscopy Sciences, Hatfield, PA). Slides were viewed, and images were collected and analyzed using a laser-scanning confocal microscope and LSM5 image browser software (LSM 510META, Zeiss, Oberkochen, Germany).

### ATP/NADH-Coupled Assay for ATPase

The ATP/NADH-coupled assay for ATPase was performed as previously described^17^,^40^. Briefly, NRK-52E cells were incubated for different times (0, 6, 12, 24, and 48 h) with prorenin (100 pmol/L). Fresh homogenates were immediately extracted from each experiment using lysis buffer, and 60 μg of protein was added to the ATPase reaction buffer for a final total volume of 150 μL (i.e., the Na^+^/K^+^-free reaction mixture). The ATPase hydrolysis rate was determined as the rate of NADH absorbance decrease at 340 nm in the Na^+^/K^+^-free reaction mixture. The ATPase reaction buffer included 25 mM triethanolaminehydrochloride (TEA) pH 7.5, 13 mM magnesium acetate, 1.8 mM DTT, 5 mM ATP, 200 g/mL BSA, 3 mM phosphoenolpyruvate (PEP), 20 U/mL pyruvatekinase/lactatedehydrogenase (PK/LDH), and 0.3 mM NADH. The lysis buffer was used as a negative control.

### V-ATPase-Mediated Proton Transport

NRK52E cells were cultured on confocal slides in 35-mm plates and treated with or without prorenin (100 pmol/L, 48 h) according to previously established methods^17^,^35^. Cells grown on confocal slides were incubated for 1 h at 37 °C in NHB (pH 7.2, in mM: 110 NaCl, 50 HEPES acid, 5 KCl, 1 MgCl_2_, 5 KH_2_PO_4_, 1 CaCl_2_, and 5 glucose) containing 10 μM SNARF-4F. Coverslips were placed on a 37 °C confocal microscope stage (LSM510, Zeiss). The monolayer was washed three times with NHB and suspended in NHB. Fluorescence intensity was measured using confocal microscopy every 5 s for 5 min at an excitation wave length of 488 nm with a slit width of 5 nm and two emission wave lengths of 580 and 640 nm with slit widths of 10 nm. Once the pHi of the cells in NHB was stable, Na-independent pHi recovery after a 20-mM NH_4_Cl-induced acid load for 5 min (NH_4_HB: similar to NHB, except 20 mM NH_4_Cl and 90 mM NaCl replaced 110 mM NaCl, pH 7.2) was determined while the cells were incubated in CHB (similar to NHB, except 110 mM choline chloride replaced 110 mM NaCl), as described previously^17^,^35^. V-ATPase-mediated pHi recovery after the acute acid load was also evaluated in the presence of the V-ATPase inhibitor bafilomycinA1 (1 nM). The fluorescence intensity ratio was calibrated to pHi at the end of each experiment using potassium HEPES buffer that contained 10 μM nigericin.

### Co-immuno precipitation

A total of 800 μg of whole cell homogenates from NRK-52E cells stimulated by prorenin (100 pmol/L, 48 h) was used for co-immunoprecipitation. Homogenates were precleared with protein A-agarose (Pierce, Rockford, IL) for 1 h at 4 °C. The supernatants were saved and incubated with a rabbit polyclonal anti-(P) RR antibody (4 μg, Abcam, Cambridge, UK) or rabbit IgG (2 μg, Sigma, MO) as a negative control at 4 °C overnight on a rotator. Protein A-agarose (50 μL, Pierce, Rockford, IL) was added to the complex and incubated for 4 h at 4 °C. The agarose pellets were washed three times with 1 mL of ice-cold lysis buffer (Cell Signaling Technology, Boston, MA) with a protease inhibitor cocktail (Cell Signaling Technology, Boston, MA) and one time in 1 mL of ice-cold PBS buffer. Bound proteins were extracted with 30 μL of 2× laemmli sample buffer (Bio-Rad) supplemented with 5% β-mercaptoethanol. The samples were heated at 95 °C for 5 min, and the supernatants were saved for Western blotting.

### Small interfering RNA for prorenin receptor and V-ATPase B2 subunit

Small interfering RNA (siRNA) oligonucleotides specifically targeting the (P) RR isoform (5′-GCUGCAUGAUAUUUCAAGUTT-3′, 5′-ACUUGAAAUAUCAUGCAGCTT-3′) ((P) RR-siRNA) and the B2 isoform (5′-CACUAGUGAUCUUAGAUCATT-3′, 5′-UGAUCUAAGAUCACUAGUGTT-3′) (B2-siRNA)^17^ and a scrambled non-targeting siRNA (siRNA-negative control) were purchased from Shanghai GenePharma (Shanghai, China). Lipofectamine 2000 (Invitrogen) was used for siRNA transfection according to the manufacturer’s instructions. Briefly, 10 μL of 20 μM siRNA and 5 μL of Lipofectamine 2000 were mixed with 250 μL of Opti-MEM (Invitrogen) and incubated for 5 min at room temperature. The siRNA/Lipofectamine 2000 mixture was incubated for 20 min at room temperature and added to an appropriate volume of DMEM, and 1 mL was then added to each 35-mm dish. Cells were incubated with siRNA for 24 h after incubation with iRNA-free media for 5-h. Cells were stimulated with prorenin for another 48 h before harvesting. Western blotting was performed as described above using the protein isolated from cells after siRNA treatment to quantify the reduction in (P) RR and the V-ATPase B2 subunit. The effects of (P) RR or V-ATPase B2 subunit silencing on prorenin-induced FN or α-SMA expression were measured using Western blotting and real-time PCR. The influence of siRNA-mediated knockdown of B2 or (P) RR on V-ATPase-mediated H^+^ transport was monitored based on Na^+^-independent pHi recovery after an acute acid load as described above. All measurements were performed in triplicate.

### Statistical analyses

All results were expressed as the means ± SD, with n representing the number of experiments. Statistical analysis was performed using standard statistical software (SPSS for Windows, version 13.0). Comparisons between multiple groups were performed using analysis of variance (ANOVA) for symmetrically distributed data and Kruskal Wallis tests for skewed distribution data. T test and Wilcoxon rank sum test were used to assess differences between two groups, as appropriate. P < 0.05 (two-tailed) was considered a statistically significant difference.

## Additional Information

**How to cite this article**: Liu, Y. *et al.* Interaction between V-ATPase B2 and (Pro) renin Receptors in Promoting the progression of Renal Tubulointerstitial Fibrosis. *Sci. Rep.* **6**, 25035; doi: 10.1038/srep25035 (2016).

## Figures and Tables

**Table 1:** Primer sequences for real-time PCR.

Gene name	Sequence	Size bp
Rat α-SMA	Forward: 5′-ACCTTCAATGTCCCTGCCATGTA-3′	227
	Reverse: 5′-ACGAAGGAATAGCCACGCTCA-3′	
Rat E-cadherin	Forward: 5′-CGTATCGGATTTGGACGGACAC-3′	220
	Reverse: 5′-GGATGGGAGCATTGTCGTTGAC-3′	
FN	Forward:5′-CTGAACCCAGTCCCGATGGTA -3′	119
	Reverse:5′-CACGTCCAACGGCATGAAG -3′	
(P) RR	Forward:5′-AGGACCATCCTTGAGACGAAACA -3′	120
	Reverse:5′-GGCCAAGCCAGTCATAATCCAC -3′	
Rat V-ATPase B2	Forward: 5′-AGCCGTGGTTCAGGTATTTG-3′	242
	Reverse: 5′-ATGCCCGTCTGAATCATCTC-3′	
Rat V-ATPase E	Forward: 5′-CCGCAAGATAAAGGTTTCCA-3′	410
	Reverse: 5′-GACAGCATGCACGACTCACT-3′	
Rat V-ATPase c	Forward: 5′-GCGGTGCTGGTATTTAGAGC-3′	401
	Reverse: 5′-GATGCCATCAGTCAGGGAGT-3′	
Rat β-actin	Forward: 5′-TGTCACCAACTGGGACGATA-3′	392
	Reverse: 5′-TCTCAGCTGTGGTGTGAAG-3′	
