# Corrigendum: Interaction between V-ATPase B2 and (Pro) renin Receptors in Promoting the progression of Renal Tubulointerstitial Fibrosis

**DOI:** 10.1038/srep27677

**Published:** 2016-06-10

**Authors:** Yun Liu, Sujun Zuo, Xiaoyan Li, Jinjin Fan, Xueqin Cao, Xueqing Yu, Qiongqiong Yang

Scientific Reports 6: Article number: 25035; 10.1038/srep25035 published online: 04282016; updated: 06102016

The Acknowledgements section in this Article is incomplete.

“Thanks very much for the contributions on statistical work by Dr Qian Zhou”.

should read:

“Thanks very much for the contributions on statistical work by Dr Qian Zhou. This work was supported by grants from the National Natural Science Foundation of China (Grant 81370786)”.

